# Expressions and clinical significances of c-MET, p-MET and E2f-1 in human gastric carcinoma

**DOI:** 10.1186/1756-0500-7-6

**Published:** 2014-01-06

**Authors:** Ju-gang Wu, Ji-wei Yu, Hong-biao Wu, Lin-hai Zheng, Xiao-chun Ni, Xiao-qiang Li, Guang-ye Du, Bo-jian Jiang

**Affiliations:** 1 1st Department of General Surgery, Shanghai 3rd People’s Hospital, School of Medicine, Shanghai Jiao Tong University, No. 280, Mohe Road, Shanghai 201900, China; 2 Department of Pathology, Shanghai 3rd People’s Hospital, School of Medicine, Shanghai Jiao Tong University, Shanghai 201900, China

**Keywords:** Stomach, Cancer/Neoplasm, c-MET, E2f-1, Ki-67, Prognosis

## Abstract

**Background:**

To investigate on the expressions and the clinical significances of hepatocyte growth factor receptor (c-MET), phosphorylated c-MET (p-MET) and e2f-1 transcription factor in primary lesion of gastric adenocarcinoma (GC).

**Method:**

Tissue samples from the primary lesion of GC in patients who accepted D_2_/D_3_ radical gastrectomy with R_0_/R_1_ resection were stained by immunohistochemistry of c-MET, p-MET, e2f-1 and Ki-67. The univariate and the multivariate analyses involving in clinicopathological parameters and prognostic factors were evaluated.

**Results:**

The positivity rates for c-MET (66.12%, 80 cases/121 cases), p-MET (59.50%, 72 cases/121 cases), e2f-1 (38.84%, 47 cases/121 cases) and Ki-67 (72.73%, 88 cases/121 cases) in primary lesion of GC was significantly higher than that in non-cancerous tissue at 5 cm places far from the margin of primary lesion (P < 0.05, respectively). The deeper tumor invasion, the severer lymph node metastasis, the later stage of TNM and the higher expression of Ki-67 was respectively an independent risk factor for the higher expression of c-MET or p-MET, but the younger age and the shorter survival time was an independent risk factor for the higher expression of e2f-1 respectively. Survival analysis showed that the worse prognosis could be observed in the patients with the combination of both c-MET-positive and e2f-1-negative (P = 0.038) or both p-MET-positive and e2f-1-negative (P = 0.042). Cox analysis demonstrated that the severer lymphatic node metastasis and the higher positivity rate of c-MET, p-MET or e2f-1 were an independent prognosis factor respectively. The higher expression of e2f-1 was identified in patients with Stage I-II, which correlated with a shorter survival time. Survival analysis also revealed that the prognosis of patients with positive expression of e2f-1 at Stage I-II was significantly worse than that in patients with negative expression of e2f-1 (*χ*^2^ = 13.437, P = 0.001). However, in the cases with Stage III-IV, no significant difference could be identified in the prognostic comparison between positive and negative expressions of e2f-1.

**Conclusions:**

The expression of c-MET or p-MET is an independent prognosis factor. It has been observed that the higher expression of e2f-1 occurred in the early stages while the lower expression of it in the later stages in GC.

## Background

Growth factors and their receptors, as factors influencing cell cycle initiation, play an important role in the initiation and the progression of tumor. The c-MET as the receptor of hepatocyte growth factor (HGF) is an important member of the growth factor receptor family, which is a protein encoded by the c-MET proto-oncogene and is a transmembrane protein mainly located at the cell membrane. The c-MET has tyrosine kinase activity and has connections with a variety of cancer gene-related products and regulatory proteins. As well known, the binding of HGF with c-MET can activate the Ras → Raf → MEK → MAPK protein signaling pathway. HGF also regulates the activation of Ras by binding with small G proteins, namely through the G protein, and is a second messenger as cAMP, cGMP, DAG, PIP3, and Ca^2+^ signaling pathway, which conveys a signal to the nucleus. This initiates or shuts down the corresponding gene transcription, leading to an increase or a decrease in the level of the cell cycle protein Cyclin D [1,2], which phosphorylates Rb to release the transcription factor e2f [3,4]. E2f-1 has been demonstrated to be a substantial member of the e2f transcription factor family relating to the cell cycle and the most important transcription activator of the Rb/e2f pathway as involved in cell cycle regulation, which regulates the transcription of the corresponding downstream gene for the transition from phase G_1_ to phase S. Because of this function, e2f-1 is considered to be essential for the proliferation and the development of cells [5].

The over-expression and gene amplification of c-met occurs during the initiation and the metastasis of many tumors [6-8]. Furthermore, the role of e2f-1 is much more complicated, having many functions of oncogenes and tumor suppressor genes. As a nuclear antigen associated with cell proliferation-specificity, the Ki-67 can be expressed in proliferating cells in all phases during cell cycle except in phase G_0_[9].

Gastric cancer is still the second cause of cancer-related death worldwide [10,11], but the mechanisms of initiation and metastasis of it have been still unclear until now. Therefore, the relation of the expression of c-MET, e2f-1 and Ki-67 with the profiles of clinicopathological parameters and prognosis in the patients with gastric adenocarcinoma (GC) would be investigated in this study of ours.

## Methods

### Clinical data

A total of 121 patients with GC, who had undergone radical surgery (D_2_ or D_3_ operation) with R_0_ or R_1_ outcome for GC from Jan 2001 to Dec 2008 in our department (male = 77 cases, female = 44 cases), were registered in this study of ours and were followed until May 31, 2011. For these cases during this period, the clinical profiles and the pathological features of them were recorded [12]. All of patients with advanced stages of GC would receive the same scheme of chemotherapy with the (8–12) regimes of 5-Fu and cisplatin after operation. Observing data such as age (62.02 years as average), tumor diameter (5.23 cm as average), metastatic lymph node ratio (35.89% as average) and survival time (20.17 months as average) were grouped with respect to the average values [13]. The information of remaining parameters were listed in Table 1. At the same time, specimens located at the places more than 5 cm far from the margin of primary lesion of cancer (so called as peri-cancer tissue) were taken and all of them were pathologically confirmed as normal gastric tissue at the same time. These samples from peri-cancer tissues were used as controls to the relatively primary foci of GC for the immunohistochemical staining [8,14,15]. This study was approved by the Ethical Committee of Shanghai 3rd People’s Hospital, School of Medicine, Shanghai Jiao-tong University before its start. The informed consent of each patient was obtained in written file from the patient or his/her relative before the patient participated in this study of ours. Written consent for publication of this study was obtained from the patients or their relative too.

**Table 1:** Expression of c-MET and its correlation with clinicopathological parameters in 121 cases

**Parameters**	**Grouping**	**n**	**c-MET [case (%)]**		***χ***^**2 **^**test**	
			**Positive**	**Negative**	***χ***^**2 **^**value**	***P *** **value**
**Age (year)**	<60	40	28(70.0)	12(30.0)	0.366	0.562
	≥60	81	52(64.2)	29(35.8)		
**Gender**	Male	77	52(67.5)	25(32.5)	1.835	0.168
	Female	44	28(63.6)	16(36.4)		
**Tumor diameter (cm)**	<5	48	36(75.0)	12(25.0)	1.108	0.216
	≥5	73	44(60.3)	29(39.7)		
**Location of tumor**	Cardia, fundus	20	17(65.0)	3(35.0)	11.239	0.003
	Body	37	30(62.2)	7(37.8)		
	Pylorus, antrum	64	33(51.6)	31(48.4)		
**Lauren classification**	Intestinal	84	60(71.4)	24(28.6)	0.823	0.132
	Diffuse	37	20(54.1)	17(45.9)		
**Histological type**	Grade 1	25	15(40.0)	10(60.0)	0.207	0.839
	Grade 2	47	31(66.0)	16(34.0)		
	Grade 3	49	34(69.4)	15(30.6)		
**Invasion depth**	T_1_	18	10(55.6)	8(44.4)	1.928	0.116
	T_2_	32	20(62.5)	12(37.5)		
	T_3_	30	21(70.0)	9(30.0)		
	T_4_	41	29(70.7)	12(29.3)		
**Metastatic lymph node ratio**	<35%	60	33(55.0)	27(45.0)	8.237	0.006
	≥35%	61	47(67.0)	14(33.0)		
**Involving lymph node station**	N_0_	43	22(51.2)	21(48.8)	9.731	0.021
	N_1_	41	30(73.2)	11(26.8)		
	N_2_	26	18(69.2)	8(30.8)		
	N_3_	11	10(90.9)	1(9.1)		
**TNM stages**	I_a_	16	9(56.2)	7(43.8)	10.722	0.015
	I_b_	17	8(47.1)	9(52.9)		
	II	20	15(75.0)	5(25.0)		
	III_a_	20	14(70.0)	6(30.0)		
	III_b_	22	16(72.7)	6(26.3)		
	IV	26	18(69.2)	8/(30.8)		
**Survival (month)**	<20	75	57(76.0)	18(24.0)	11.337	0.001
	≥20	46	23/(50.0)	23/(50.0)		

### Experimental reagents

The rabbit-derived monoclonal antibodies against human such as anti-c-MET (D1C2; Cell Signaling TECHNOLOGY, Beverly, MA), anti-c-MET phosphorylation (p-MET) (Tyr1234/1235; D26; Cell Signaling TECHNOLOGY, Beverly, MA), anti-e2f-1 (USCN Life Science Ltd Co., Wuhan, China) and anti-Ki-67 (USCN Life Science Ltd Co., Wuhan, China) were used. The biotinylated secondary antibody was a goat anti-rabbit antibody (Changdao Co., Shanghai, China). The kit of streptococcus avidin-biotin-peroxidase complex (SABC) method and the DAB chromogenic reagent were purchased from Changdao Co., Shanghai, China.

### Immunohistochemical staining procedure

The immunohistochemical staining method was used by SABC method, and the procedure was based on the product specifications. All tissue samples from primary foci and the peri-cancer tissue (at 5 cm to the margin of tumor as normal tissue checked by pathological observation) were fixed with 10% formalin, routinely embedded in paraffin, cut into 4-μm thick as serial sections, dewaxed with xylene, rehydration with an ethanol gradient, soak-treated with 0.3% hydrogen peroxide solution, washed with distilled water and placed in sodium citrate buffer. The microwave heating method was adopted for antigen retrieval. The samples were washed with PBS, blocked with bovine serum and then incubated overnight at 4°C with the primary antibody (1:100 dilutions). Biotinylated second antibody was added, and the samples were incubated for 30 min at room temperature. The samples were washed with PBS to remove any unbound antibody. SABC reagent was added, and then the samples were incubated for 20 min at room temperature, washed with PBS, and stained with DAB. Next the samples were washed with distilled water, re-stained with hematoxylin. Finally, the samples were dehydrated and made transparent, sealed and examined with a microscope. PBS was used as a negative control instead of the primary antibody. A sample provided by the company with known positive results was used as a positive control.

### Determination of the positive results

Two pathological researchers who were blind to the corresponding clinical and pathological data read the slices. It was necessary to have the same results read by these two researchers. If the different opinion for their observation on the same sample occurred in these two pathological researchers, another senior pathologist would observe this sample and then make the final decision on this observation. The evaluation criteria were as follows: a) Brown-yellow was positive; five high power fields were randomly selected for each slice and 500 cells were counted. b) Positive rating: slices containing less than 5% positive cells were considered as negative expression, and slices with greater or equal to 5% positive cells were considered as positive expression. In summary, 5% ~ 25%, 26% ~ 50%, and more than 50% positive cells were respectively classified as weak, moderate and strong positivity.

### Statistical analysis

SPSS 13.0 software (SPSS Inc., Chicago, IL) was used for our analysis. A chi-square test was carried out to analyze the relation of the expressions of c-MET, p-MET, e2f-1 and Ki-67 with the clinicopathological parameters respectively. Two-category logistic regression analysis based on multiple parameters was adopted to deduce the relationship between the expression of c-MET, p-MET and e2f-1 and each parameter respectively. Univariate chi-square analysis and bivariate Spearson relative analysis were applied among the expression of c-MET, p-MET, e2f-1 and Ki-67. The Kaplan-Meier method and a log-rank test were used to draw and to compare the survival curves, respectively. The Cox regression model for multiple parameters influencing prognosis and the K-Related Samples test were used to compare the average survival times in different groups. P values less than 0.05 were considered statistically significant.

## Results

**Figure 1:**
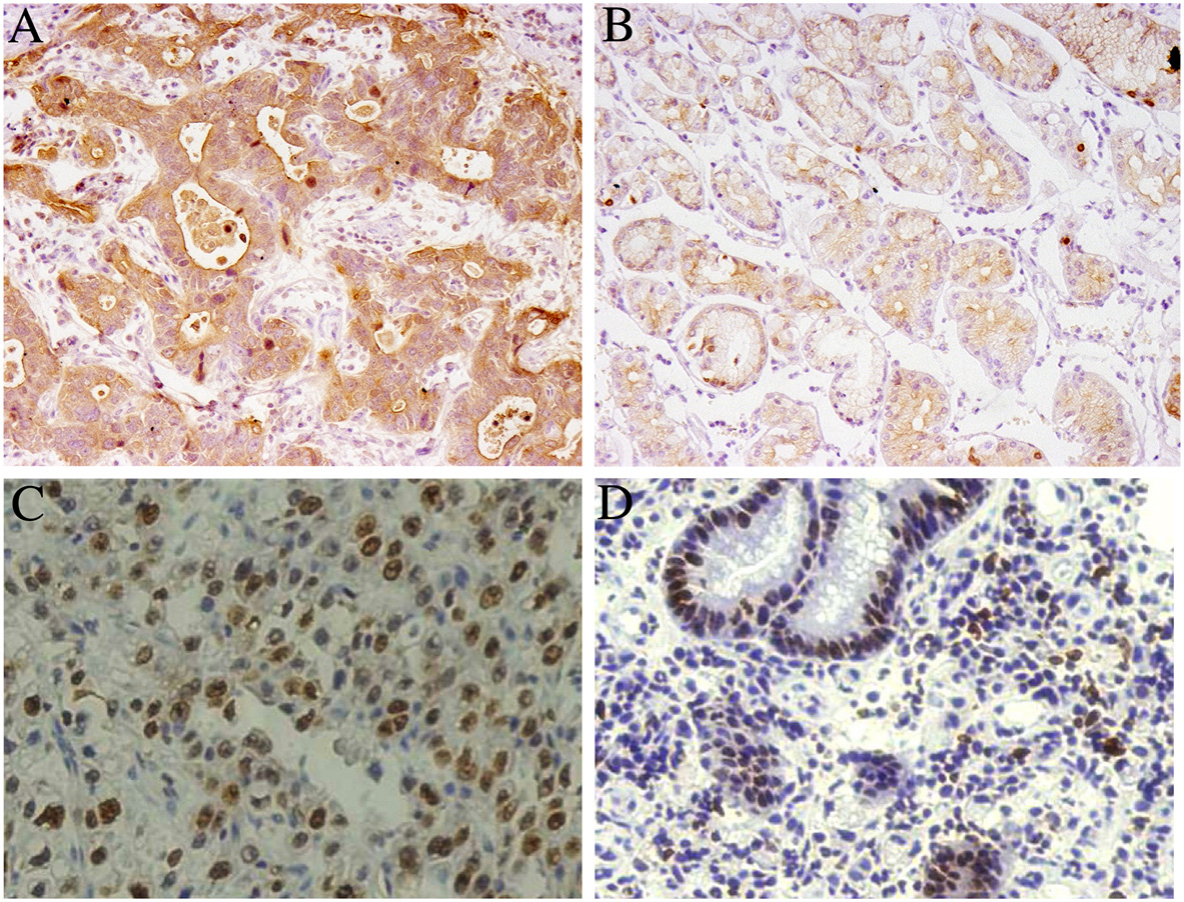
**Immunohistochemical staining for the primary foci of GC (SABC method).** Notes: **A**: c-MET; **B**: p-MET, **C**: Ki-67; **D**: e2f-1.

Tumor cells exhibiting brown granules were considered positive. To be specific, the nuclear staining was primarily observed for Ki-67, the membrane and cytoplasmic staining were observed for c-MET and p-MET, and the positive particles were observed in cytoplasm or nuclei for e2f-1 (Figure 1). Overall, 66.12% (80 cases/121 cases) of c-MET, 59.50% (72 cases/121 cases) of p-MET, 38.84% (47 cases/121 cases) of e2f-1 and 72.73% (88 cases/121 cases) of Ki-67 as the positivity rates in primary lesion were significantly higher than 34.71% (42 cases/121 cases; P = 0.001), 32.23% (39 cases/121 cases; P = 0.001), 14.88% (18 cases/121 cases; P = 0.005) and 29.75% (36 cases/121 cases; P = 0.002) in the non-cancerous tissue respectively.

### Relationship of expression of c-MET, p-MET and e2f-1 with clinicopathological characteristics

The significantly higher positivity rate of c-MET expression was respectively identified in the groups of the tumor located in upper part of stomach (*P* = 0.003), the ≥ 35% of metastatic lymph node ratio (P = 0.006), the severer lymph node metastasis (P = 0.021), the later stages of TNM (P = 0.015) and the ≥ 20 months of average survival time (P = 0.001) in comparison with the lower positivity rate of c-MET expression in the groups of the tumor located in the lower and middle parts of stomach, the < 35% of metastatic lymph node ratio, the milder lymph node metastasis, the earlier stages of TNM and the < 20 months of average survival time (Table 1).

The significantly higher positivity rate of p-MET expression was respectively found in the groups of the tumor located in upper part of stomach (*P* = 0.009), the ≥ 35% of metastatic lymph node ratio (P = 0.007), the severer lymph node metastasis (P = 0.042), the later stages of TNM (P = 0.016) and the ≥20 months of average survival time (P = 0.004) in comparison with the lower positivity rate of p-MET expression in the groups of the tumor located in the lower and middle parts of stomach, the < 35% of metastatic lymph node ratio, the milder lymph node metastasis, the earlier stages of TNM and the < 20 months of average survival time (Table 2).

**Table 2:** Expression of p-MET and its correlation with clinicopathological parameters in 121 cases

**Parameters**	**Grouping**	**n**	**p-MET [case (%)]**		***χ***^**2 **^**test**	
			**Positive**	**Negative**	***χ***^**2 **^**value**	***P *** **value**
**Age (year)**	<60	40	24(60.0)	16(40.0)	0.766	0.862
	≥60	81	48(59.3)	33(40.7)		
**Gender**	Male	77	48(62.3)	29(37.7)	1.976	0.187
	Female	44	24(54.5)	20(45.5)		
**Tumor diameter (cm)**	<5	48	33(68.8)	15(31.3)	1.138	0.236
	≥5	73	39(53.4)	34(46.6)		
**Location of tumor**	Cardia, fundus	20	15(75.0)	5(25.0)	10.252	0.009
	Body	37	27(73.0)	10(27.0)		
	Pylorus, antrum	64	30(46.9)	34(53.1)		
**Lauren classification**	Intestinal	84	55(65.5)	29(34.5)	0.924	0.186
	Diffuse	37	17(45.9)	20(54.1)		
**Histological type**	Grade 1	25	13(52.0)	12(48.0)	0.192	0.741
	Grade 2	47	28(59.6)	19(40.4)		
	Grade 3	49	31(63.3)	18(36.7)		
**Invasion depth**	T_1_	18	9(50.0)	9(50.0)	1.996	0.156
	T_2_	32	18(56.3)	14(43.8)		
	T_3_	30	19(63.3)	11(36.7)		
	T_4_	41	26(63.4)	15(36.6)		
**Metastatic lymph node ratio**	<35%	60	30(50.0)	30(50.0)	8.556	0.007
	≥35%	61	42(68.9)	19(31.1)		
**Involving lymph node station**	N_0_	43	20(46.5)	23(53.5)	9.651	0.042
	N_1_	41	27(65.9)	14(34.1)		
	N_2_	26	16(61.5)	10(38.5)		
	N_3_	11	9(81.8)	2(18.2)		
**TNM stages**	I_a_	16	8(50.0)	8(50.0)	10.887	0.016
	I_b_	17	8(47.1)	9(52.9)		
	II	20	13(65.0)	7(35.0)		
	III_a_	20	13(65.0)	7(35.0)		
	III_b_	22	14(63.6)	8(36.4)		
	IV	26	16(61.5)	10/38.5)		
**Survival (month)**	<20	75	52(69.3)	23(30.7)	10.006	0.004
	≥20	46	20/(43.5)	26/(56.5)		

However, the significantly higher positivity rate of e2f-1 expression was respectively observed in the groups of the tumor with ≥ 5 cm (P = 0.001), the ≥ 35% of metastatic lymph nodes ratio (P = 0.013), the deeper invasion of tumor (P = 0.003), the later stages of TNM (P = 0.017) and the ≥ 20 months of average survival time (P = 0.005) in comparison with the lower positivity rate of e2f-1 expression in the groups of the tumor with < 5 cm, the < 35% of metastatic lymph node ratio, the slighter invasion of tumor, the earlier stages of TNM and the < 20 months of average survival time (Table 3).

**Table 3:** Expression of e2f-1 and its correlation with clinicopathological parameters in 121 cases

**Parameters**	**Grouping**	**n**	**e2f-1 [case (%)]**		***χ***^**2 **^**test**	
			**Positive**	**Negative**	***χ***^**2 **^**value**	***P *** **value**
**Age (year)**	<60	40	17(42.5)	23(57.5)	0.336	0.562
	≥60	81	30(37.0)	51(63.0)		
**Gender**	Male	77	32(41.6)	45(58.4)	0.657	0.418
	Female	44	15(34.1)	29(65.9)		
**Tumor diameter (cm)**	<5	48	27(56.3)	21(43.7)	10.148	0.001
	≥5	73	20(23.4)	53(76.6)		
**Location of tumor**	Cardia, fundus	20	7(35.0)	13(65.0)	0.226	0.893
	Body	37	14(37.8)	23(62.2)		
	Pylorus, antrum	64	26(60.6)	38(59.4)		
**Lauren classification**	Intestinal	84	36(42.9)	48(57.1)	1.863	0.172
	Diffuse	37	11(29.7)	26(70.3)		
**Histological type**	Grade 1	25	10(40.0)	15(60.0)	0.236	0.889
	Grade 2	47	17(36.2)	30(63.8)		
	Grade 3	49	20(40.8)	29(59.2)		
**Invasion depth**	T_1_	18	12(66.7)	6(33.3)	14.143	0.003
	T_2_	32	17(53.1)	15(46.9)		
	T_3_	30	7(23.3)	23(76.7)		
	T_4_	41	11(26.8)	30(73.2)		
**Metastatic lymph node ratio**	<35%	60	30(50.0)	30(50.0)	6.237	0.013
	≥35%	61	17(27.9)	44(72.1)		
**Involving lymph node station**	N_0_	43	21(48.8)	22(51.2)	6.739	0.081
	N_1_	41	17(41.5)	24(58.5)		
	N_2_	26	8(30.8)	18(69.2)		
	N_3_	11	1(9.1)	10(90.91)		
**TNM stages**	I_a_	16	10(62.5)	6(37.5)	13.724	0.017
	I_b_	17	8(47.1)	9(52.9)		
	II	20	11(55.0)	9(45.0)		
	III_a_	20	8(40.0)	12(60.0)		
	III_b_	22	6(27.3)	16(72.7)		
	IV	26	7(26.9)	19(73.1)		
**Survival (month)**	<20	75	52(69.3)	23(30.7)	10.356	0.005
	≥20	46	20/(43.5)	26/(56.5)		

### Relationships among the expressions of c-MET, p-MET and e2f-1

The higher positivity rate of c-MET was significantly correlated with the higher positivity rate of p-MET expression (P = 0.001) or Ki-67 expression (P < 0.001) respectively. And the higher positivity rate of p-MET was significantly correlated with the higher positivity rate of Ki-67 expression (P < 0.001). By relative analysis, the positive expression of c-MET was positively related to the positive expression of p-MET (P = 0.001). And the positive expression of either c-MET (P < 0.001) or p-MET (P < 0.001) was positively related with the positive expression of Ki-67 respectively also (Table 4).

**Table 4:** Chi-square analysis and Spearson relative analysis among the expression of c-MET, p-MET, e2f-1 and Ki-67

**Parameters**		**c-MET expression (n/%)**					
		**Positive**	**Negative**	***χ***^**2 **^**value**	***P***_***1 ***_value^*^	**r value**	***P***_***2 ***_**value**^**#**^
**c-MET**	**Positive**	80/66.1					
	**Negative**		41/33.9				
**P-MET**	**Positive**	70/57.9	2/1.7	28.765	0.001	0.635	0.001
	**Negative**	10/8.3	39/32.2				
**e2f-1**	**Positive**	33/27.3	14/11.6	0.766	0.290	0.142	0.395
	**Negative**	47/38.8	27/22.3				
**Ki-67**	**Positive**	70/57.8	18/14.9	28.262	<0.001	0.621	<0.001
	**Negative**	10/8.3	23/19.0				
		**p-MET expression (n/%)**					
**e2f-1**	**Positive**	29/24.0	18/14.9	0.811	0.308	0.195	0.518
	**Negative**	43/35.5	31/25.6				
**Ki-67**	**Positive**	66/54.5	22/18.2	29.233	<0.001	0.667	<0.001
	**Negative**	6/5.0	27/22.3				
		**e2f-1 expression (n/%)**					
**Ki-67**	**Positive**	35/28.9	53/43.8	0.021	0.858	-0.018	0.861
	**Negative**	12/9.9	21/17.4				

Note: *: Chi-square analysis, #: Spearson relative analysis.

### Logistic analysis regarding to the expressions of c-MET, p-MET and e2f-1

Multivariate analysis revealed that the deeper tumor invasion [P = 0.017, 95.0% CI. for Exp (B) = 0.000 ~ 0.368], the severer lymph node metastasis [P = 0.010, 95.0% CI. for Exp (B) = 0.000 ~ 0.253], the later stage of TNM [P = 0.009, 95.0% CI. for Exp (B) = 4.390 ~ 354.32] and the higher expression of Ki-67 [P = 0.001, 95.0% CI. for Exp (B) = 2.870 ~ 66.900] were the independent risk factors for the higher expression of c-MET respectively.

At the same time, the multivariate analysis showed that the deeper tumor invasion [P = 0.025, 95.0% CI. for Exp (B)= 0.000 ~ 0.468], the severer lymph node metastasis [P = 0.021, 95.0% CI. for Exp (B) = 0.000 ~ 0.358], the later stage of TNM [P = 0.011 95.0% CI. for Exp (B) = 3.390 ~ 302.37] and the higher expression of Ki-67 [P = 0.034, 95.0% CI. for Exp (B) = 2.898 ~ 54.943] were the independent risk factors for the higher expression of p-MET respectively.

However, the younger age [P = 0.011, 95.0% CI. for Exp (B) = 0.897 ~ 0.986] and the shorter survival time [P = 0.028, 95.0% CI. for Exp (B) = 0.933 ~ 0.996] were the independent risk factors for the higher expression of e2f-1 respectively as suggested by the multivariate analysis.

### Survival analysis

#### Analysis on prognosis

**Figure 2:**
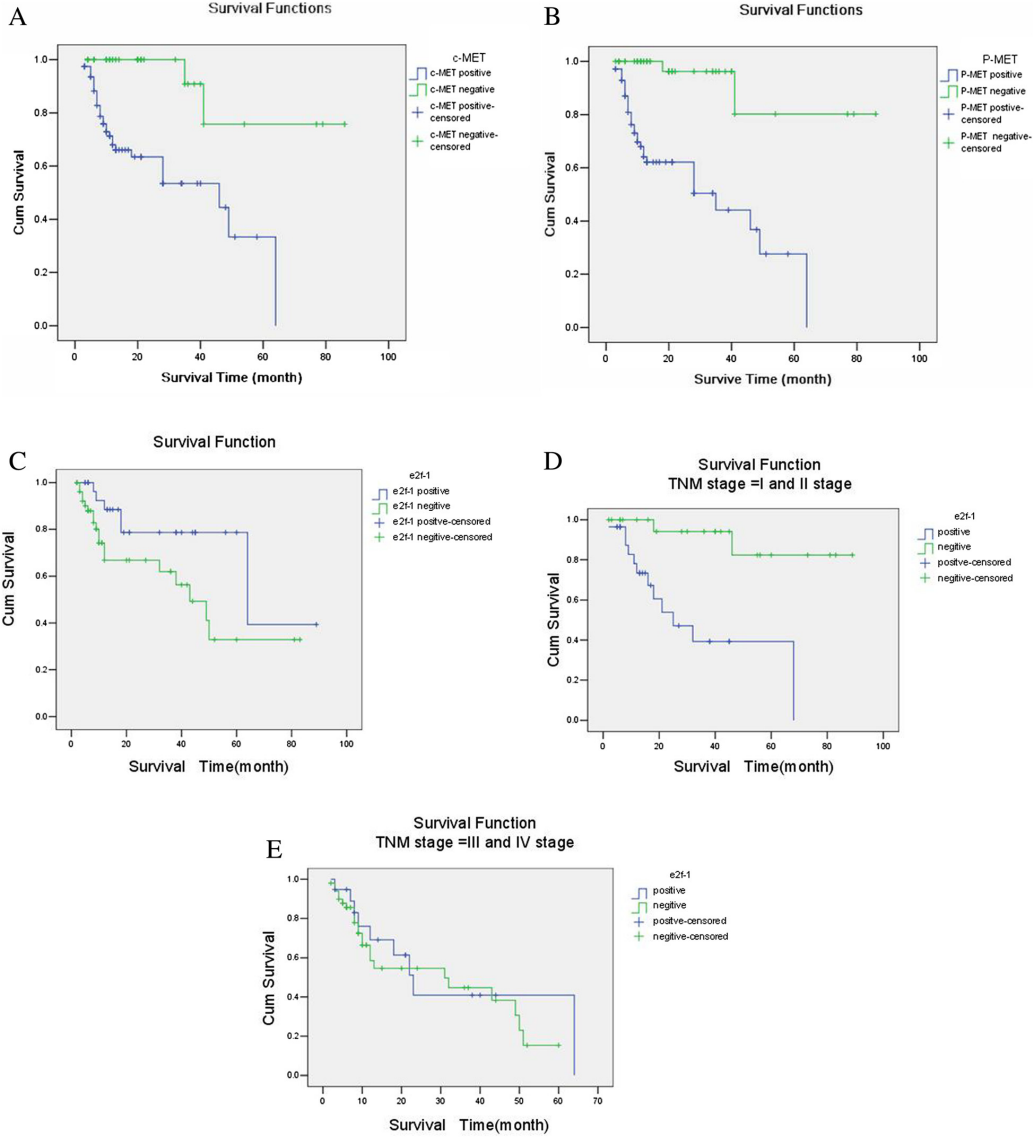
**Postoperative long-term survival curves regarding to the expression of c-MET, p-MET and e2f-1.** Notes: **A**: Grouped by c-MET expression (P = 0.001); **B**; Grouped by p-MET expression (P = 0.001). **C**: Grouped by e2f-1 expression (P = 0.036). **D**: In patients with stages I and II grouped by e2f-1 positive and negative expression; **E**: In patients with stages III and IV grouped by e2f-1 positive and negative expression.

As shown in Table 5, log-rank analysis of prognosis demonstrated that the correlated factors with worse prognosis included the ≥ 5 cm tumor in diameter (P = 0.002), the tumor located in cardia, fundus and body of stomach (P = 0.017), the deeper invasion of tumor (P = 0.008), the ≥35% of metastatic lymph nodes ratio (P = 0.003), the severer lymph nodes metastasis (P < 0.001), the later stages of TNM (P < 0.001), the higher positivity rate of c-MET expression (P = 0.001) (Figure 2A), the higher positivity rate of p-MET expression (P < 0.001) (Figure 2B), the lower positivity rate of e2f-1 expression (P = 0.036) (Figure 2C) and the higher positivity rate of Ki-67 expression (P = 0.002). Survival analysis also revealed that the worse prognosis was observed in the patients with the combination of both c-MET-positive and e2f-1-negative (P = 0.038) or both p-MET-positive and e2f-1-negative (P = 0.042). As shown by Cox stepwise regression model analysis (items to observe included in the standard univariate analysis P < 0.05), the severer lymph nodes metastasis [P < 0.001, 95.0% CI for Exp (B) = 1.504 ~ 2.747] and the higher positivity rate of c-MET expression [P = 0.038, 95.0% CI for Exp (B) = 1.152 ~ 3.005], p-MET expression [P = 0.035, 95.0% CI for Exp (B) = 1.005 ~ 2.981] or e2f-1 expression [P = 0.032, 95.0% CI for Exp (B) = 1.058 ~ 3.602] shared the worse prognosis respectively. As revealed by the Cox model analyses for the relationship of prognosis with the positivity rates of 4 factors, 3 factors and 2 factors in combination with c-MET, p-MET, e2f-1 and Ki-67, the positivity of all 4 factors was the independently prognostic factor [P = 0.003, 95.0% CI for Exp (B) = 1.033 ~ 4.323] and the positivity of 3 factors of p-MET, e3f-1, Ki-67 was also the independently prognostic factor [P = 0.012, 95.0% CI for Exp (B) = 1.015 ~ 4.023]. But the positivity in other combination of these factors was not independently prognostic factor. However, other variate such as tumor location, tumor size, invasion depth, TNM stage and higher expression of Ki-67 was not the independently prognostic factors too.

**Table 5:** Survival analysis on all case by Log-rank analysis (n = 121)

**Parameters**	**Grouping**	**Case number (n/%)**	***χ***^**2 **^**value**	**df**	***P *** **value**
**Tumor diameter (cm)**	<5	48/(39.7)	9.190	1	0.002
	≥5	73/(60.3)			
**Tumor location**	Cardia, fundus	20/(16.5)	8.122	2	0.017
	Body	37/(30.6)			
	Pylorus, antrum	64/(52.9)			
**Invasion depth**	T_1_	18/(14.9)	11.712	3	0.008
	T_2_	32/(26.4)			
	T_3_	30/(24.8)			
	T_4_	41/(33.9)			
**Involving lymph node station**	N_0_	43/(35.5)	24.017	3	<0.001
	N_1_	41/(33.9)			
	N_2_	26/(21.5)			
	N_3_	11/(9.1)			
**Metastatic lymph node ratio**	<35%	60/(49.6)	8.596	1	0.003
	≥35%	61/(50.4)			
**TNM stage**	I_a_	16/(13.2)	25.405	5	<0.001
	I_b_	17/(14.0)			
	II	20/(16.5)			
	III_a_	20/(16.5)			
	III_b_	22/(18.2)			
	IV	26/(21.5)			
**c-MET expression**	Positive	80/(66.1)	12.084	1	0.001
	Negative	41/(33.9)			
**p-MET expression**	Positive	72(59.5)	5.687	1	<0.001
	Negative	49(40.5)			
**e2f-1 expression**	Positive	47/(38.8)	3.217	1	0.036
	Negative	74/(61.2)			
**Ki-67 expression**	Positive	88/(72.7)	9.348	1	0.002
	Negative	33/(27.3)			

#### Connection of c-MET, p-MET or e2f-1 subgroup regarding to positivity rate with prognosis

Analysis on subgroup of either c-MET positive expression or p-MET positive expression revealed that the average survival month of the strongly positive group [(15.1 ± 2.9) mo, P = 0.002] was significantly shorter than that in the moderately [(22.1 ± 3.3) mo.] and weakly [(29.8 ± 3.8) mo.] positive groups, and simultaneously, that the average survival month of the strongly positive group [(16.5 ± 4.1) mo, P = 0.003] was significantly shorter than that in the moderately [(23.0 ± 2.9) mo.] and weakly [(30.1 ± 4.1) mo.] positive groups. However, the analysis of e2f-1 expression subgroup demonstrated that the average survival month of the strongly positive group [(15.4 ± 2.7) mo., P = 0.017] was also significantly shorter than that in the moderately [(22.5 ± 2.9) mo.] and weakly [(26.2 ± 2.8) mo.] positive groups.

#### Stratified analysis on prognosis

Performing the correlation of e2f-1 expression with prognosis among patients with different TNM stages of GC generated the results as follows: the total positivity rate of e2f-1 expression was 41.3% (50 cases/121 cases), and the positivity rates for Stage I_a_, I_b_, II, III_a_, III_b_ and IV were 62.5% (10 cases/16 cases), 47.1% (8 cases/17 cases), 55% (11 cases/20 cases), 40% (8 cases/20 cases), 27.3% (6 cases/22 cases) and 26.9% (7 cases/26 cases), respectively. These results showed that e2f-1 was expressed at a higher level in patients with Stage I-II, and that the higher expression of e2f-1 correlated with a shorter survival time in the early stage of GC. On the other hand, lower expression of e2f-1 was observed in patients with Stage III-IV, but no connection between e2f-1 expression and survival time was observed in patients with Stage III-IV based on statistical analysis (Table 6). Survival rate analysis by log-rank showed that the prognosis of patients positive for the expression of e2f-1 at Stage I-II was significantly worse than that in patients negative for the expression of e2f-1 (*χ*^2^ = 13.437, P = 0.001). However, such difference for the prognosis of patients between the positive and negative for the expression of e2f-1 was not significant among patients during Stage III-IV (*χ*^2^ = 0.283, P = 0.595) (Figure 2D and 2E).

**Table 6:** Stratified survival analysis of e2f-1 expression in groups with different TNM stage

**TNM groups**	**Grouping (month)**	**e2f-1 expression (n/%)**		***χ***^**2 **^**value**	***P***_***1 ***_value^*^	***χ***^**2 **^**value**	***P***_***2 ***_**value**^**#**^
		**Positive**	**Negative**				
**Stage I-II**	< 20	19/36.5	9/17.3	4.792	0.029	-0.304	0.029
	≥ 20	9/17.3	15/28.8				
**Stage III-IV**	< 20	10/14.5	37/53.6	2.895	0.081	0.205	0.091
	≥ 20	9/13.0	13/18.8				

Notes: *: Log-rank analysis; #: K-Related Samples test.

## Discussions

### Requirements for molecular classification about prognostic improvements

To choose the optimal treatment in order to improve the prognosis of GC, it is necessary to seek more sensitive indicators for the early diagnosis and much more exquisite choice for the optimal treatment. Such indicators as tumor invasion depth, the number of regional lymph node metastases, distant metastasis and histological grade were used in UICC classification [16], but the different catalog in stations for regional lymph nodes according to tumor location and the different stage based on some comprehensive evaluation such as liver metastasis, peritoneal metastasis and ascites cytology were applied in JGCA [12]. Both of these two classifications aimed at evaluating diseases status more accurately, choosing treatments more appropriate and making evaluations more precise on therapeutic efficacy. Because of the the complexity and the diversity in the initiation, development and metastasis of tumor as revealed by evidences of molecular biology, it was found that both lymph node metastasis and the abnormal expression of a variety of biological molecules in the primary tumors and the metastatic foci of GC could lead to the different prognosis in GC [8,13-15,17]. As revealed in this study of ours, although severer lymph node metastasis was the independently prognostic factor, the TNM stage could not be identified as the independently prognostic factor, which might be due to the occurrences of deviations in both of TNM staging and N staging (i.e., lymph node staging), as well as in micro-metastasis of lymph node [13,17]. Therefore, the molecular classification applying the merits of tumor markers in molecular biology in GC might be of some supplements to overcome the deviation and the faultinesses of either UICC classification or JGCA classification.

### Regulation of c-MET, p-MET and e2f-1

Tumor cells are characterized by abnormal cell cycle initiation coupled with unrestricted proliferation. The start of the cell cycle is inseparable from growth factors. If growth factors are lacking during the G_1_ phase, the cell cycle will stop at the restriction point and the cell will pass into the quiescent state, namely the G_0_ phase. G_0_ phase cells enter G_1_ phase when stimulated by growth factors. The binding of a growth factor with its receptor induces a signal which activates Cyclin D-Cdk4/6 and phosphorylates Rb, thereby releasing the e2f transcription factor, which is mainly made up of e2f-1. This process in turn gives rise to the transcription of several genes, driving the cell cycle to continue and mediating the transition from phase G_1_ to phase S [18].

The c-MET binding to HGF causes the growth, the proliferation and the differentiation of the target cell. The incremental expression of c-MET has been identified in hepatocellular carcinoma, gastric carcinoma and colon carcinoma [7,19,20]. Extensive expression of c-MET, especially p-MET as activated c-MET, in GC was identified during this research of ours. Furthermore, either c-MET or p-MET is more often highly expressed in cases with tumor located in the upper part of stomach, larger than 35% of metastatic lymph node ratio, severer lymph node involvement, <20 months of survival, later stage of TNM and stronger proliferation of cells (higher expression of Ki-67). Thus, it could be proposed that either c-MET or p-MET was a useful marker of the malignant behavior of GC and an effective factor for the prognosis of GC. However, it is necessary for these more precise conclusions to do the further investigations.

Recently, the relationship between e2f-1 and apoptosis of tumor cells, which plays a complicated role in the generation and progression of many kinds of tumors, has received more attention as revealed by experimental study [21]. E2f-1 is able to bind with the promoter of the insulin-like growth factor-I receptor (IGF-IR) gene in prostate cancer cells to promote the transcription of IGF-IR, thus enhancing the proliferation of tumor cells [22]. The over-expression of e2f-1 in non-small cell lung cancer can activate the expressions of thymidylate synthase and survivin, and then encourage the proliferation of tumors, finally resulting in the worse prognosis [23]. On the other hand, some reports have proposed that e2f-1 might suppress cancer cells. For the elucidation to this phenomenon, e2f-1 can bind directly to promoter of the BIN1 tumor-suppressor gene when DNA is damaged, then trans-activating BIN1 to induce apoptosis. E2f-1 can also activate the gene Cancer 1, another tumor-suppressor gene whose promoter is methylated [24,25]. Our univariate analysis from the samples of human GC showed that e2f-1 was more highly expressed in patients at Stage I and II. Furthermore, multivariate analysis coupled with TNM-stratified analysis revealed that patients who were positive for e2f-1 expression at the cases with Stage I and II had worse prognoses. Thus, this outcome to seem of contradiction hinted that e2f-1 might be a key transcription factor which stimulated the conversion from normal cells to cancer cells; therefore, it might be why its expression was markedly lower in the later periods of progression of GC.

E2f-1 can induce apoptosis in many types of tumor cells, which has been demonstrated by in vitro gene transfection experiments [26]. Enhanced invasion of advanced GC might be related to the decreased expression of e2f-1, which might transcriptionally regulate down several tumor-suppressing genes and apoptosis-related genes. However, many pathways could not regulate oncogenes. Therefore, tumor cell proliferation and survival were further out of control. As such, e2f-1 might potentially inhibit tumor cells and shed new light on the choice of gene therapy for patients with late-stage of tumors after revealing in detail on the more clear mechanism of this regulation.

Based on classical signaling pathways, c-MET is an upstream molecule to e2f-1. E2f-1 should be regulated by p-MET as the active status of c-MET, but we could not find such connection either between c-MET and e2f-1 (r = 0.142, P = 0.395) or between p-MET and e2f-1 (r = 0.195, P = 0.518). This indicates that in addition to being a target molecule of c-MET, e2f-1 may be regulated by some other signaling pathways, which is worthy of making the further investigations on this regulation of signaling pathway. Nevertheless, when the expression of e2f-1 was lower during the later stages of advanced GC, c-MET and p-MET were still highly expressed. Therefore, further investigation for the relationship of p-MET with e2f-1 is required to verify this hypothesis in clinical and experimental studies.

## Conclusion

The expression of either c-MET or its activity status, i.e. p-MET, is one of the markers reflecting the degree of malignancy of GC, and either c-MET or p-MET is an effective prognosis factor because the significantly shorter survival time of patients positive for expression of c-MET and p-MET. E2f-1 was highly to express in relatively earlier stages of GC, while the lower expression of e2f-1 was observed in later periods of GC. As such, e2f-1 could play different roles at different stages of cancer; that is, it might promote the formation of the cancer in the early stages but inhibit the progression of cancer in the intermediate and advanced stages.

## Abbreviations

GC: Gastric adenocarcinoma; HGF: Hepatocyte growth factor; p-MET: Phosphorylated c-MET; PBS: Phosphorylation buffer solution; SABC: Streptococcus avidin-biotin-peroxidase complex; UICC: The United States Joint Committee on Cancer; JGCA: The Japanese Gastric Cancer Association.

## Competing interests

The authors declare that they have no competing interests.

## Authors’ contributions

JGW and JWY contributed in study design, editing and revision of manuscript. HBW and JGW contributed in study concepts, carried out study design, definition of intellectual content, literature research, experimental studies, data acquisition, data analysis, statistical analysis and manuscript preparation. JWY, JGW, LHZ, XCN and BJJ contributed in clinical managements. XQL and GYD contributed in pathological studies. BJJ contributed in guarantor of integrity of the entire study, study concepts, study design, manuscript review and revised version correction. BJJ and JWY contributed in the grants for this study. All authors read and approved the final manuscript.

## Authors’ information

JWY and LHZ are associated professors of surgery and JWY is acting chairman of 1st Department of General Surgery in our hospital. JGW and XCN are doctor-in-charges of 1st Department of General Surgery in our hospital. HBW was a postgraduate student in our hospital and works as doctor-in-charge of surgery in Wu-gang People’s Hospital, Hubei Province now. XQL is associated professor of pathology and GYD is lecturer of pathology. BJJ is a professor of surgery and vise chairman of Department of General Surgery in our hospital.
